# A Case of Lyme Carditis With Variable Heart Block Successfully Treated With Oral Doxycycline

**DOI:** 10.7759/cureus.24729

**Published:** 2022-05-04

**Authors:** Ian Del Valle, Victor Hoang, Stuart T Wood

**Affiliations:** 1 Internal Medicine, Keesler Medical Center, Biloxi, USA; 2 Infectious Disease, Keesler Medical Center, Biloxi, USA

**Keywords:** ekg abnormalities, first degree atrioventricular block, second degree heart block, iv ceftriaxone, oral doxycycline, ixodes scapularis tick, tick-borne infections, carditis, lyme disease and other tick borne pathogens, lyme's disease

## Abstract

A 39-year-old male without significant past medical history presented with three weeks of worsening fatigue, migratory arthralgia, rash, and unilateral facial weakness after spending three months in Vermont. Serology showed positive Lyme titers 1:64 for both IgM and IgG. EKG on presentation showed a P-R interval of 384 ms, and the patient was admitted for concern of Lyme carditis. Serial EKGs obtained throughout his stay demonstrated variability between first- and second-degree heart blocks. After consultation with Infectious Disease, he was transitioned to oral doxycycline to complete a 21-day course. The patient’s heart block and other symptoms had resolved on follow-up after the treatment course had been completed.

## Introduction

Lyme disease, a spirochetal infection caused by members of the genus Borrelia, is the most common vector-borne disease in the United States [1]. From 2008 to 2019, it is estimated that around 1% of all cases in the United States consisted of Lyme carditis [2]. The pathophysiology of Lyme carditis is poorly understood as it is usually not fatal and cardiac biopsies are not routinely performed [3]. Cardiac features of Lyme disease can be seen with early disseminated Lyme disease and have been documented to occur in a range from <1 week to >28 weeks after onset of infection. These can occur simultaneously with other manifestations of the disease. Atrioventricular (AV) conduction block can occur. It can alternate between the degree of the block within minutes, with the highest risk of progressing to third-degree AV block in those with a P-R interval over 300 ms [4]. Patients with a P-R interval of greater than 300 ms, second degree, or third-degree heart block warrant an admission to start antibiotic therapy as heart block can become fatal [5]. Guidelines have recommended IV ceftriaxone for a P-R interval greater than 300 ms; however, many studies and case reports discussed later suggest oral doxycycline is non-inferior in treating disseminated Lyme disease, including Lyme Carditis.

This article was previously presented as a meeting abstract at the 2021 Mississippi ACP Abstract Day on September 30, 2021.

## Case presentation

**Figure 1:**
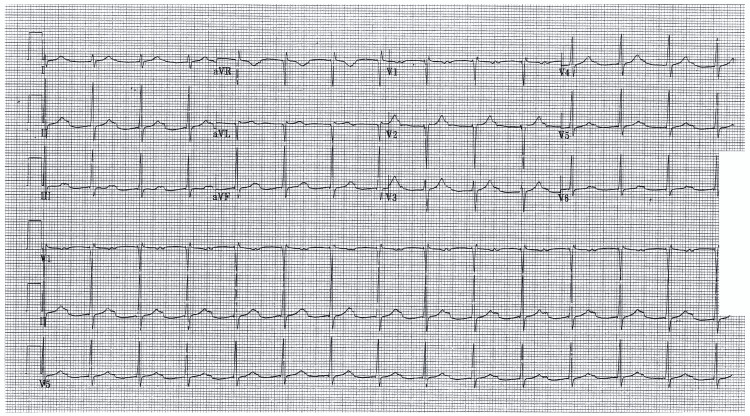
Hospital day 1 ECG with first-degree atrioventricular block.

A 39-year-old male without significant past medical history presented to the ED with three weeks of worsening fatigue and unilateral facial weakness. The patient had recently moved to Mississippi for an extended stay for military training. Previously, he worked as a tennis pro for three months in the summer at a resort in Vermont. While in Vermont, the patient stated he spent a significant part of his downtime hiking in the woods and swimming in nearby lakes. He denied any animal bites, bug bites, ticks on skin, cave diving, drug use, over-the-counter supplementation, chemical exposures, and had been sexually abstinent for more than one year. He had noticed a small, symmetric rash on his left inner thigh around three weeks prior to presentation. He described the rash as flat, circular, non-erythematous, non-tender, and without central clearing. He stated that it had been resolved a few days prior to presentation to our ED. He also noted right-sided facial weakness, including an inability to raise his right eyebrow, around three weeks prior to the presentation, which had slowly resolved over the subsequent weeks. One week prior to the presentation, he developed left-sided facial weakness, including the inability to raise his left eyebrow, which was still present at the time of presentation. He endorsed migratory arthralgia starting on the right temporomandibular joint and eventually involving his left hip and knee over the course of two weeks. This arthralgia had fully resolved by his time of presentation. On examination, the patient was hemodynamically stable, lying-in bed comfortably, and in no acute distress. His cardiopulmonary exam was unremarkable with regular rate and rhythm, with no murmurs, gallops, or rubs. His abdomen was soft, non-tender, and non-distended. He had no focal neurological deficits other than his left-sided facial weakness. An ECG was obtained, showing a P-R interval of 384 ms (Figure 1).

**Figure 2:**
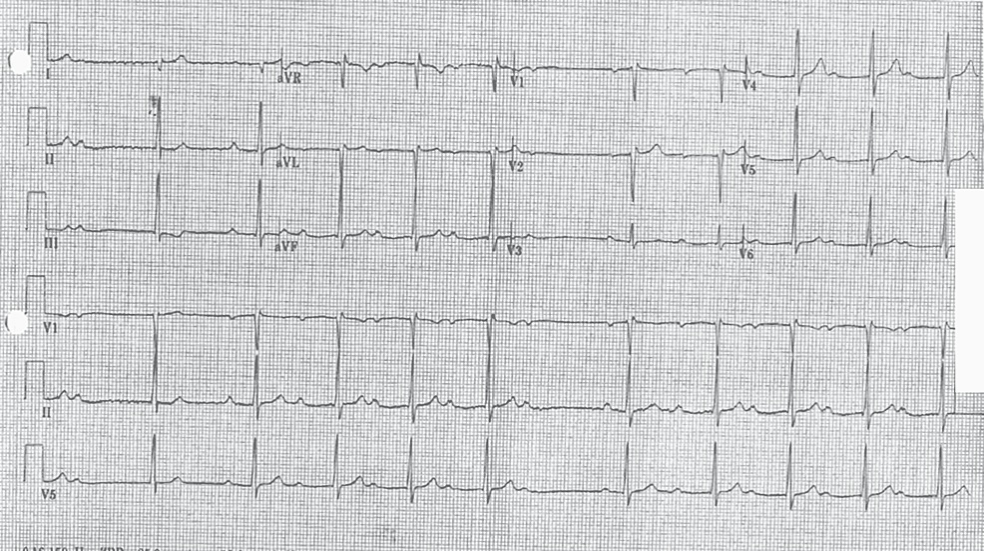
Hospital day 2 ECG with second-degree, Mobitz type one atrioventricular block.

Given that his PR interval was longer than 300 ms, and there was a clinical suspicion of Lyme carditis, he was admitted to the hospital for antibiotic therapy and serial EKGs. He was started on 2 g of ceftriaxone daily and monitored on telemetry. Though this diagnosis was primarily obtained based on the clinical presentation, serologic testing was performed to confirm the diagnosis, and other etiologies were considered. Lyme serologies were positive at 1:64 for both IgG and IgM; given these results, a Western blot test was not necessary. The following morning EKG showed that he had progressed to second-degree type one heart block (Figure 2).

An ECG was ordered to evaluate for structural abnormalities. Transthoracic ECG showed no structural or functional abnormalities with a 55-60% ejection fraction. On the evening of hospital day 2, EKG showed a return to first-degree AV block. After a review of the literature and with infectious diseases input, it was decided to complete therapy with oral doxycycline rather than continuing IV ceftriaxone. On hospital day 3, the morning EKGs showed second-degree, Mobitz type one heart block, so the patient was discharged on 21 days of doxycycline 100 mg twice a day with close follow-up. EKG at follow-up after antibiotic course completion showed heart block had resolved with a P-R interval of 184 ms with the resolution of other symptoms as well.

## Discussion

This case represents a presentation of Lyme carditis with a severely prolonged P-R interval that progressed to second-degree type 1 AV block, successfully treated with oral doxycycline. Current guidelines suggest the use of IV ceftriaxone to treat Lyme carditis, but this is based on expert opinion [6]. When possible, we would like to avoid using long-term IV antibiotics as this is an inconvenience to the patient, and there are possible complications to long-term IV access. Transitioning to oral antibiotics also allows the patient to be treated at home instead of in the hospital.

We conducted a literature review analyzing the safety of using oral doxycycline in place of IV ceftriaxone for the treatment of Lyme carditis. We started with case reports that described similar cases to ours in which they were discharged on oral doxycycline and reported a good outcome [7]. We then explored existing studies that compared IV ceftriaxone to oral doxycycline in disseminated Lyme. One study by Dattwyler RJ et al. compared parenteral ceftriaxone to oral doxycycline for acute disseminated Lyme disease without meningitis [8]. This study enrolled 140 patients and found that both regimens were well-tolerated and were equally effective at preventing late manifestations of Lyme disease. In another trial, Kortela E et al. looked at 210 patients with Lyme neuroborreliosis, treated with either oral doxycycline or parenteral ceftriaxone [9]. This study did a subjective analysis of symptoms one year out from initiating treatment and found no difference between the two groups. We found no studies suggesting ceftriaxone was superior to doxycycline in the treatment of Lyme carditis.

Given our findings, when reviewing existing literature, we felt it was appropriate to transition to oral doxycycline for 21 days and follow up as an outpatient. Our patient had complete resolution of symptoms using this approach to treatment. If our patient had developed a higher degree of atrioventricular block requiring pacing, our treatment approach might have changed. However, our patient never developed a higher degree block than second-degree type I after two nights on telemetry and serial EKGs. Therefore, we felt that our patient was safe for outpatient management given this information.

## Conclusions

Lyme carditis is a rare manifestation of Lyme disease, with a variable clinical course and uncertain treatment strategy. Physicians encountering Lyme carditis should understand the indications for inpatient admission with IV antibiotics and use clinical judgment to determine the transition to oral antibiotics and outpatient management.
